# Rimonabant improves metabolic parameters partially attributed to restoration of high voltage-activated Ca^2+^ channels in skeletal muscle in HFD-fed mice

**DOI:** 10.1590/1414-431X20176141

**Published:** 2017-05-04

**Authors:** B. Chen, N. Hu

**Affiliations:** 1 Department of Orthopedics, Zhongnan Hospital, Wuhan University, Wuhan, Hubei Province, China; 2 Department of Concurrent Chemoradiation Lymphatic Hematopoietic Comprehensive Ward, Zhongnan Hospital, Wuhan University, Wuhan, China

**Keywords:** Cannabinoid receptor type 1, Obesity, Rimonabant, Skeletal Muscle, HVA calcium channel

## Abstract

Cannabinoid type 1 receptor (CB1R) inhibition tends to be one of the promising strategies for the treatment of obesity and other related metabolic disorders. Although CB1R inhibition may cause adverse psychiatric effects including depression and anxiety, the investigation of the role of peripheral CB1R on weight loss and related metabolic parameters are urgently needed. We first explored the effect of rimonabant, a selective CB1R antagonist/inverse agonist, on some metabolic parameters in high fat-diet (HFD)-induced obesity in mice. Then, real-time PCR and electrophysiology were used to explore the contribution of high voltage-activated Ca^2+^ channels (HVACCs), especially Ca_v_1.1, on rimonabant's effect in skeletal muscle (SM) in HFD-induced obesity. Five-week HFD feeding caused body weight gain, and decreased glucose/insulin tolerance in mice compared to those in the regular diet group (P<0.05), which was restored by rimonabant treatment compared to the HFD group (P<0.05). Interestingly, HVACCs and Ca_v_1.1 were decreased in soleus muscle cells in the HFD group compared to the control group. Daily treatment with rimonabant for 5 weeks was shown to counter such decrease (P<0.05). Collectively, our findings provided a novel understanding for peripheral CB1R's role in the modulation of body weight and glucose homeostasis and highlight peripheral CB1R as well as Cav1.1 in the SM as potential targets for obesity treatment.

## Introduction

Insulin resistance (IR) tends to be an important predictor for various metabolic disorders, including obesity. Skeletal muscle (SM) is a principal peripheral tissue in maintaining glucose metabolism and in the development of IR, accounting for the majority of total insulin-stimulated glucose uptake. Fully understanding the mechanisms for glucose uptake in SM remains a hot topic for the investigation of IR and metabolic diseases.

The importance of Ca^2+^ signaling in IR and related metabolic disorders has been well established. A rapid elevation in intracellular Ca^2+^ has been reported to regulate glucose transporter type 4 (GLUT4) traffic and increase surface GLUT4 level (1), which is associated with glucose uptake in muscle and fat cells. Jang and other researchers have demonstrated that decrease of the concentration of free intracellular Ca^2+^ by a Ca^2+^ chelator restored glucose infusion rate in SM in a high-fat diet (HFD) rat model (2,3).

Accumulating clinical studies have indicated that chronic administration of rimonabant (SR141716, a selective antagonist/inverse agonist of cannabinoid type 1 receptor (CB1R)), significantly reduces body weight and improves glycemic control and lipids in obese patients with type 2 diabetes (4–6). CB1Rs are widely expressed in the brain, adipose tissue, liver, pancreas and skeletal muscle, which are all closely associated with metabolic regulation (7–9). *In vitro* studies have shown that reduced insulin-stimulated glucose uptake by adipocyte-conditioned medium is completely prevented by rimonabant in human skeletal muscle cells (7). CB1Rs tend to be a promising target for the management of type 2 diabetes. However, the mechanism that mediates the regulation of CB1R on glucose uptake in SM remains unclear.

Rimonabant has been shown to increase glucose uptake in the isolated soleus muscle of obese mice (10). A recent study has revealed that activation of protein kinase A (PKA) and phosphatidylinositol-3-kinase (PI3K) signaling accounts for rimonabant-induced glucose uptake elevation in SM cells (11). Potentiation of L-type high voltage-activated Ca^2+^ channels (HVACCs) by glucagon-like peptide-2 (GLP-2) has been revealed in a PKA-dependent manner, which contributes to glucose uptake by primary cultured hippocampal neurons (12). Considering the key role of HVACCs in Ca^2+^ signaling regulation and the importance of Ca^2+^ signaling to obesity, we hypothesized that CB1 receptor antagonists against body weight gain and improves glucose homeostasis, which is at least partly attributed to the restoration of HVACCs downregulation in skeletal muscle by HFD feeding.

## Material and Methods

### Animals

All experimental procedures were approved by the Institutional Animals Care and Use Committee of Wuhan University of China and adhered to International Animal Welfare Legislation and Rules. A total of 39 male C57BL/6J mice (6 weeks old) were used in this study. The mice were housed under a 12-h light/dark cycle (lights on at 7:00 am) and fed the HFD (40% fat, Teklad Custom Research Diet, TD 95217; Harlan, USA) or regular diet (6.5% fat, #2920; Harlan, USA).

### Chronic rimonabant treatment

Rimonabant or vehicle (0.1% Tween 80 in saline) was administered to mice at a daily dose of 30 mg/kg body weight (13) by oral gavage for 5 weeks. Body weight was monitored once a week.

### Intraperitoneal glucose tolerance test (IPGTT)

**Figure 1:**
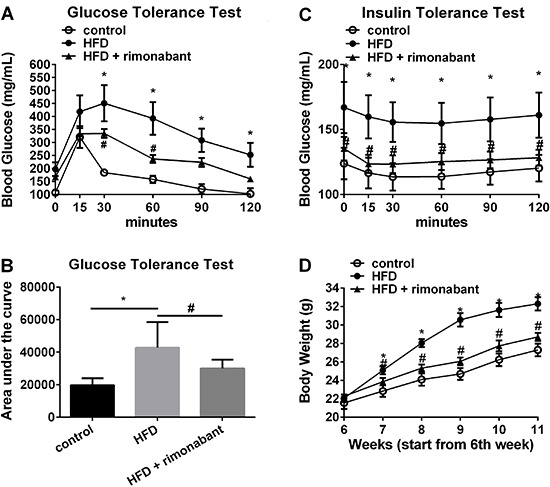
*A*, Glucose tolerance in mice fed a regular diet (control, n=6 mice), a high-fat diet (HFD, n=6 mice), or HFD+rimonabant (n=10) (*A* and *B*). *C*, Insulin tolerance test results for control (n=4 mice), HFD (n=4 mice) and HFD+rimonabant (n=4 mice) groups. *D*, Body weight of control (n=10 mice), HFD (n=10 mice) and HFD+rimonabant groups (n=14 mice) over a 5-week period. Results are reported as means±SE. *P<0.05 HFD *vs* control, ^#^P<0.05 HFD *vs* HFD+rimonabant (*A*, *C* and *D,* two-way ANOVA with Tukey's multiple comparisons. *B*, one-way ANOVA).

After 5 weeks on HFD, the mice were fasted overnight and then received *ip* injections of D-glucose (2 g/kg) prior to initiation of the glucose tolerance test modified according to a previous description (14). Blood glucose was measured from a tail venous puncture at 0, 15, 30, 60, 90, and 120 min (Figure 1) using a glucometer. The area under the glucose tolerance curve was analyzed.

### Intraperitoneal insulin tolerance test (IPITT)

Like the glucose tolerance test, after a 6-h fast, mice received *ip* injections of insulin (1 U/kg) prior to initiation of the insulin sensitivity test (14). Blood was drawn at serial time points for blood glucose measurement as described above.

### Soleus muscle cells preparation

Primary soleus muscle cells were cultured similar to a previously study (15), with modifications. The mice from the IPGTT or IPITT were used. Briefly, mice were deeply anesthetized with isoflurane and the soleus was removed from the hind legs of mice. The soleus was quickly placed in ice-cold growth medium (GM) containing Dulbecco's modified Eagle's medium: 4.5 g/L glucose, 4 mM L-glutamine, 50 U/mL penicillin, 50 µg/mL streptomycin, and 20% fetal bovine serum. The soleus muscle was minced into small pieces and forced through the tip of a 10-mL pipette, and then incubated in 5 mL GM (serum replaced by 195 U/mL collagenase type I) for 3 h at 37°C. Individual cells were dissociated by triturating the tissue through a fire-polished glass pipette and centrifuged at 300 *g* for 5 min at room temperature. After centrifuging 3 times, the cells were planted on poly-D-lysine pre-coated glass culture dishes (15 mm diameter) in GM with 20% fetal bovine serum at 37°C in a water saturated atmosphere with 5% CO_2_.

### Whole-cell patch-clamp recording

Spherically shaped cells were selected for whole-cell patch clamp recording, using an Axopatch 200B amplifier (Axon Instruments, USA) and the output was digitized with a Digidata 1332A converter and pClAMP 9 software (Axon Instruments). Data were acquired at a sampling rate of 2 KHz. Data obtained from cells in which uncompensated series resistance resulted in voltage-clamp errors >5 mV were discarded. To measure the HVACCs in soleus muscle cells, cells were held at -80 mV and depolarized from -50 mV to +40 mV for 450 ms, in 10 mV increments with 5-s intervals. The amplitude of HVACCs was calculated as peak negative current.

A recording microelectrode with a tip resistance of 2-4 MΩ was filled with the pipette solution: 120 mM CsCL, 0.1 mM CaCL_2_, 2.0 mM MgCL_2_, 10.0 mM EGTA, 10.0 mM HEPES and 5.0 mM Tris-ATP, pH adjusted to 7.2 with CsOH. The external solution contained: 110 mM Choline-Cl, 20 mM TEACl, 5 mM BaCl_2_, 2.0 mM MgCl_2_, 10 mM HEPES, and 20 mM D-glucose, adjusted to pH 7.4 with CsOH. Ba^2+^ was used as the charge carrier when recording HVACCs.

### Real-time PCR analysis of Ca_v_1.1 expression in soleus muscle

Twenty-four hours after IPGTT or IPITT, sac and soleus muscles were immediately collected from the mice and frozen at -80°C. Total mRNA from the soleus muscle was extracted using Trizol (Invitrogen, USA). cDNA was prepared from 1 µg of RNA using SuperScript III First-Strand cDNA Synthesis kit (Invitrogen). Quantitative real-time PCR assay was performed according to published protocols (16). The primer pair for Ca_v_1.1 was as follows: Ca_v_1.1-F, GTTACATGAGCTGGATCACACAG; Ca_v_1.1-R, ATGAGCATTTCGATGGTGAAG. The relative mRNA level of Ca_v_1.1 in each sample was first normalized to the level of the housekeeping gene β-actin, which was 1.

### Chemicals

Cell culture materials were purchased from GIBCO (Life Technologies, USA). Rimonabant was purchased from TOCRIS (Tocris Cookson, UK). All other chemicals, unless otherwise stated, were from Sigma (USA).

### Statistical analysis

Data are reported as means±SE. Statistical analyses were carried out with SigmaPlot 12 (USA). The data were compared using one-way ANOVA with Duncan's *post hoc* test, two-way ANOVA with Turkey's *post hoc* test, or paired Student's *t*-test. P<0.05 was considered to be significant.

## Results

### HFD feeding caused obesity

Five weeks after the assigned diet, HFD-fed mice showed a significant increase in body weight, compared with control mice (10 mice in each group, P<0.05, Figure 1D).

### HFD feeding decreased glucose and insulin tolerance

Blood glucose levels during the IPGTT were significantly higher in HFD-fed mice, compared with control mice (6 mice in each group, Figure 1A), as evidenced by the increased area under the curve for the glucose tolerance test (P<0.05, Figure 1B). In line with a previous study (17), HFD-fed mice showed an impaired insulin tolerance (Figure 1C).

### CB1 antagonist reduced HFD-induced obesity

In line with the anti-obesity effect of rimonabant reported before (18,19), 5-week daily administration of rimonabant (n=14) caused a significant body weight loss in HFD-fed mice, compared to untreated HFD-fed mice (P<0.05, Figure 1D).

### CB1 antagonist improved metabolic parameters in HFD-fed mice

HDF mice that received a five-week daily administration of rimonabant displayed an improved glucose tolerance (10 mice for HFD+rimonabant group) and insulin tolerance (4 mice for HFD+rimonabant group, Figure 1A–C).

### CB1 antagonist restored decreased Ca_v_1.1 expression in soleus muscle in HFD-fed mice

**Figure 2:**
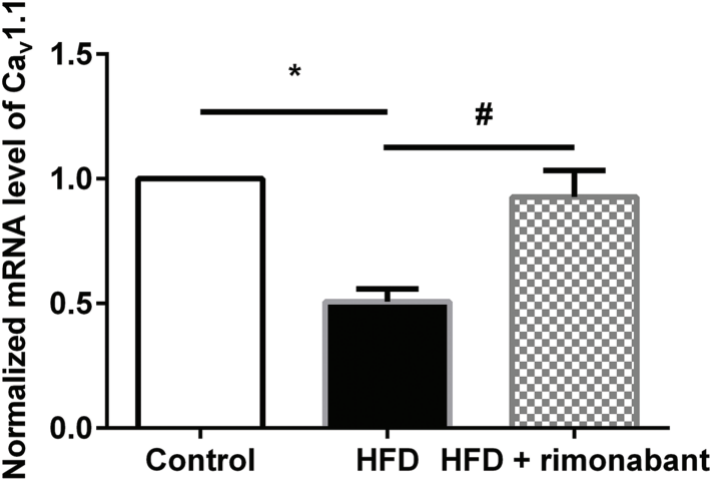
Expression levels of Ca_v_1.1 in soleus muscle in regular diet (control; n=5), high-fat diet (HFD) and HFD+rimonabant groups. Data are reported as means±SE. *P<0.05, HFD *vs* control, ^#^P<0.05, HFD *vs* HFD+rimonabant (repeated measures one-way ANOVA was used).

Real-time PCR revealed that the level of Ca_v_1.1 in soleus muscle was decreased in HFD-fed mice, compared with control mice. Rimonabant increased Ca_v_1.1 levels in soleus muscle in HFD-fed mice, compared to the untreated HFD group (Figure 2).

### CB1 antagonist restored decreased HVACC in SM cells in HFD-fed mice

**Figure 3:**
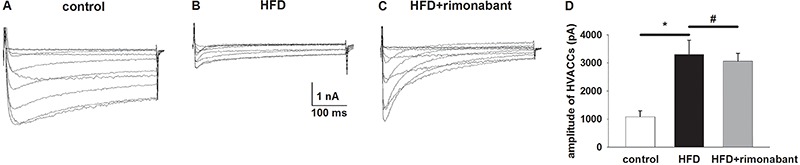
Changes in the amplitude of high voltage-activated calcium channels (HVACCs) in soleus muscle cells from regular diet (control, *A*), HFD diet (*B*) and HFD+rimonabant (*C*) groups. *D*, Summary data of HVACCs amplitude in the three groups. Data are reported as means±SE. *P<0.05, regular *vs* HFD; ^#^P<0.05, HFD *vs* HFD+rimonabant (one-way ANOVA followed by Duncan's *post hoc test*).

The amplitude of HVACCs was significantly decreased in soleus muscle cells in HFD-fed mice compared with control mice (P<0.05). Furthermore, a 5-week rimonabant treatment significantly restored decreased HVACCs in HFD-fed mice (P<0.05, Figure 3).

## Discussion

In the present study, we found that mice on a 5-week HFD had a body weight gain, and decreased glucose tolerance as well as insulin tolerance, which were restored by rimonabant treatment. Furthermore, HFD feeding decreased the expression levels of Ca_v_1.1 and the function of HVACCs in SM cells, which was restored by rimonabant treatment. Our findings support our hypothesis that CB1R blockade restores decreased HVACCs in SM in HFD-fed mice, which at least partly contributes to CB1R blockade induced-protection of body weight control and glucose homeostasis in diet-induced obesity.

Accumulating evidence from clinical trials suggests that CB1R blockade plays a protective role in controlling body weight and glucose homeostasis in humans (20–23). Rimonabant, a brain and peripheral CB1R antagonist/inverse agonist, is one of the most recent drugs designed to treat obesity and its use was approved in Europe in 2006. We found that a 5-week rimonabant treatment reduced body weight gain induced by a HFD in mice. Furthermore, rimonabant treatment improved glucose homeostasis in HFD-fed mice, as evidenced by the results of IPGTT and IPITT.

However, rimonabant may cause adverse psychiatric effects including depression and anxiety (24–28). For this reason, rimonabant was rejected by the Food and Drug Administration in the United States and withdrawn from the European market in early 2009. Nevertheless, it is still too early to end hope for CB1R inhibition in obesity treatment. There is evidence that the neutral CB1R-selective antagonist without intrinsic activity (29), AM4113, has been reported to have anti-obesity effects at doses that do not induce symptoms of nausea and vomiting (29,30). One of the most promising approaches is to develop CB1R antagonists/inverse agonists selectively acting on peripheral CB1R, and thus lacking psychiatric side effects (31–33). In this regard, the understanding of the peripheral effects of CB1R on obesity is urgently needed. Ca_v_1.1 E1014K knock-in mice (EK) are generated with a Ca^2+^ binding and/or permeation defect in Ca_v_1.1, which shows blocked Ca^2+^ binding, decreased Ca^2+^ influx, and decreased CaMKII activity (34). EK mice display increased body weight and impaired glucose and insulin tolerance relative to wide type mice (35). These findings have important implications on the promising effects of Ca_v_1.1 in controlling body weight and glucose homeostasis. To test the peripheral effects of rimonabant on obesity, the changes of expression level of Ca_v_1.1 in SM were detected in different feeding conditions including regular diet, HFD diet and in HFD diet+rimonabant treatment. We found that the HFD decreased the expression level of Cav1.1 in SM, and 5-week rimonabant treatment restored this decrease. Ca_v_1.1 is one of the major HVACCs in skeletal muscles. HVACCs are decreased in coronary arteries smooth muscle in HFD feeding (36). However, the effect of HVACCs in SM cells in HFD feeding and rimonabant treatment is unclear. Using whole cell patch clamp, we found that HFD decreased the functional HVACCs as evidence by decreased amplitude of HVACCs in SM cells, which was also restored by rimonabant. Thus, our findings suggest that HVACC, especially Ca_v_1.1 in SM cells, is a promising peripheral target for CB1R antagonist obesity protection.

Rimonabant is an antagonist/inverse agonist for CB1R. Whether rimonabant restores the effects of HFD on body weight and glucose homeostasis as an antagonist or as an inverse agonist is controversial. There is evidence that the neutral CB1R-selective antagonist without intrinsic activity (29), AM4113, shares the ability of the CB1R antagonist/inverse agonist to suppress body weight gain as we reported here and in others reports (30,37). In the contrary, other authors argue that CB1 inverse agonist reduces food intake and body weight (38,39). Further, as an inverse agonist, rimonabant also possibly acts on calcium channels and in HVACCs increase (40). Rimonabant appears to become a neutral antagonist at the K192A mutant CB1R. Thus, the increased HVACCs in rimonabant treatment compared to the decreased HVACCs in HFD mice may be due to the blockage of rimonabant on CB1R activation-induced inhibition on HVACCs as an antagonist. Thus, it is necessary to better understand the role of CB1R antagonist or inverse agonist on anti-obesity effects. In summary, we provided evidence that peripherally targeting CB1R and its action on HVACCs, especially Ca_v_1.1 in the SM, could be therapeutically advantageous for obesity treatment.
